# Personalizing the BioPsychoSocial Approach: “Add-Ons” and “Add-Ins” in Generalist Practice

**DOI:** 10.3389/fpsyt.2021.716486

**Published:** 2021-11-24

**Authors:** William B. Ventres, Richard M. Frankel

**Affiliations:** ^1^Department of Family and Preventive Medicine, University of Arkansas for Medical Sciences, Little Rock, AR, United States; ^2^Regenstrief Institute, Indiana University School of Medicine, Indianapolis, IN, United States

**Keywords:** biopsychosocial models, general practitioners, medical education, medical philosophy, physician-patient relations, primary care, systems theory

## Abstract

Generalist practitioners often find interacting with patients deeply satisfying and joyful; they also experience encounters that are challenging and complex. In both cases, they must be aware of the many issues that affect the processes and outcomes of patient care. Although using the BioPsychoSocial approach is an important, time-tested framework for cultivating one's awareness of patients' presenting concerns, recent developments suggest that additional frames of reference may enhance communication and relationships with patients. In this article, we describe several additions to the BioPsychoSocial approach, considerations we call “add-ons” and “add-ins”. We invite generalist practitioners and, indeed, all health care practitioners, to consider how they can improve their ongoing care of patients by personalizing these and other additions in their day-to-day work with patients.

## Introduction

Based on systems theory and later complemented by patient- and relationship-centered care, the BioPsychoSocial (BPS) approach has become an important part of medical practice and medical education, especially among generalist practitioners (1–4). The approach is applicable as a way of conceptualizing, organizing, and addressing the physical, emotional, and social factors that influence how patients experience and describe their presenting concerns. Although not without its critics (5–7), and certainly not limited to generalist practice alone (8–10), the BPS approach has become one of the most important—some might suggest *the* most important—unifying model in generalist medicine over the last four decades (11).

Much has changed in generalist medicine since mention of the value of systems theory to patient care and publication of the seminal paper on the BPS approach (12, 13), which appeared in *Science* in 1977 (1). Significantly, scholars have established the overall importance of generalist principles and practices to highly functioning health care systems and improved population-based health care outcomes (14, 15). Much has changed in respect to the BPS approach, as well. From its origins as a theoretical approach to patients presenting principally with Somatic Disorders [now also referred as Medically Unexplained Symptoms (MUS) (16), Bodily Distress Syndrome (BDS) (17), or the patient-centered acronym PRESSS (Physical Reaction to Emotional Stress of Some Sort (18))], the BPS approach has sequentially emerged as a key element in both Patient-Centered Medicine and Relationship-Centered Care (19–21). The approach has also found adherents beyond generalist practice, and clinicians in a wide variety of specialties and sub-specialties (as well as many other health care professionals) have spoken to its utility in attending to patient concerns (8–10, 22, 23). Research into the BPS approach has evolved significantly over the years, and many evidence-based and evidence-informed studies have confirmed its benefits in clinical practice (24–26).

Nonetheless, much remains the same. Due to cultural and economic forces within medicine that prioritize site-specific technological interventions and highly-remunerative patterns of practice over holistic approaches to patient care (especially in countries that are highly dependent on for-profit models of health care, such as United States), generalism has struggled to find its place as a foundational element of medical education and practice (27). Additionally, theories that undergird such practice, including the BPS approach, continue to languish in the shadows of the dominant, strictly biomedical understanding of medicine. Even among generalist practitioners, the BPS approach remains undervalued relative to the more traditional linear methods of diagnosis and treatment (28). In many educational institutions, the BPS approach is manifestly far from being fully implemented; it is unmistakably given lip service, glanced over, or simply ignored in the face of a biomedically-focused pedagogical paradigm (26).

What can be done? Motivated by our (1) mutual misgivings regarding the traditional enculturation of medical students and residents away from thinking holistically and systemically, (2) recognition of the importance of the BPS approach to generalist practice, and (3) firm belief of the approach's positive influence on patients' health, we suggest it is time to reconsider how generalist practitioners understand and use the approach. We base our considerations on 70 years' combined direct clinical experience in and research observations of generalist practice—one of us is a seasoned family physician/medical anthropologist (WV) and the other a veteran medical sociologist/medical educator (RF)—plus a growing literature that speaks to the importance of the BPS approach and its successors on quality of care, (29) overall outcomes (24–26), and interpersonal satisfaction (30).

We frame our considerations in two opposing directions: first, as add-ons to the BPS approach—ways to expand our appreciation of patient-oriented concerns; and, second, as add-ins—ways to appreciate the approach as a means of influencing our own cognitive habits and practice behaviors. The purpose of this article, thus, is to help generalist practitioners personalize their use of the BPS approach so as to help nurture their therapeutic presence with patients and, ultimately, positively influence patients' health.

## Adding-on to the BPS Approach

The traditional BPS approach refers to a natural system hierarchy in which patients are located somewhere on a continuum between subatomic particles and the biosphere (1, 2). The BPS approach suggests clinicians focus on the level of patients as people first, simultaneously appreciating how other system constituent themes influence patients' experience of disease and illness. From a thematic perspective, the traditional approach focuses, self-evidently, on the biological, psychological, and social dimensions of patients' lives.

Over the years, clinicians and scholars have added-on several other themes to the original approach. Some years ago, “spiritual” became a common appendage in generalist literature, giving recognition to the influence of religion and spirituality on the health and well-being of human beings (31, 32). As well, cultural and political-economical themes of care emerged as early generalist add-ons (5, 33). Much more recently, a number of other add-ons have come to the fore from outside of generalist circles—examples include such auxiliary themes as history, microhistory, and intersectionality (from psychiatry) (34, 35), social changeways and dynamic microsystems (from psychology) (36, 37), and institutional influences (from physiotherapy) (38).

Our personal favorite thematic add-ons, broad in scope echoing our generalist backgrounds, are ecological and existential in nature. We do not, however, recommend anyone use the term “BioPsychoEcoSocialExistential.” It is a quite a mouthful and, simply, another artificial construct with extra perceptual boundaries to contend with. We prefer generalists keep things simple—BioPsychoSocial is perfectly suitable in this regard (Table 1).

**Table 1:** BioPsychoSocial add-ons: ecological and existential themes.

**Theme**	**Rationale**
Ecological	The ecological theme is informed by the environments in which people live and the influence these environments exert on individual and collective health and well-being. Ecological considerations are myriad and affect spaces large and small. On a macro scale, they include such factors as the effects on health and well-being of natural and built environments (39); the significance of both geographic community of origin and the effects of local, regional, and global migration (40); the distinct influences of rural, suburban, and urban living (41); and the looming burden of climate change (42). The appearance of COVID-19 as a global pandemic, the emergence of the Me Too and Black Lives Matter movements, and the end of the US occupation of Afghanistan are three recent events that have already and will likely further change the macro-ecological dimensions of lived environments around the world. An example of the ecological dimension on a micro scale is the migration of the Electronic Medical Record from the back room to the examination room or hospital suite (43). Although documenting while doctoring has transformed medicine in many advantageous ways (e.g., medication reconciliation, order entry, and access to internet resources), it has also posed significant challenges (44). By splitting attention between the computer and the patient, it has triggered unintended consequences that often lead to “distracted doctoring” (45, 46). In addition, taking ecological influences on health into account can be as simple as acknowledging where patients present: in an ambulatory clinic, community hospital emergency department, or academic medical center (47).
Existential	The existential dimension of the BPS approach focuses attention how patients make meaning in the face of disease and illness and how practitioners reciprocally bear witness to and experience their patients' suffering (48, 49). There is clearly a spiritual aspect to these dimensions, as some scholars have already noted and named as the “BioPsychoSocialSpiritual” model (31, 32). Meaning, however, transcends spirituality (50). Work, relationships, community, education, awareness, and ethical considerations are also sources of meaning for patients, whether practiced as behaviors, habits of mind, or soulful ways of being. So, too, are instances of intersubjective awareness, moments of “connection” when doctors and patients harmoniously recognize and acknowledge each other's humanity at a very basic level (51). From the perspective of traditional practice, in which generalists routinely meet their patients and those who accompany them in clinic examination rooms or hospital suites, these moments play key roles in establishing a shared presence that is therapeutic in and of itself (52).

**Figure 1:**
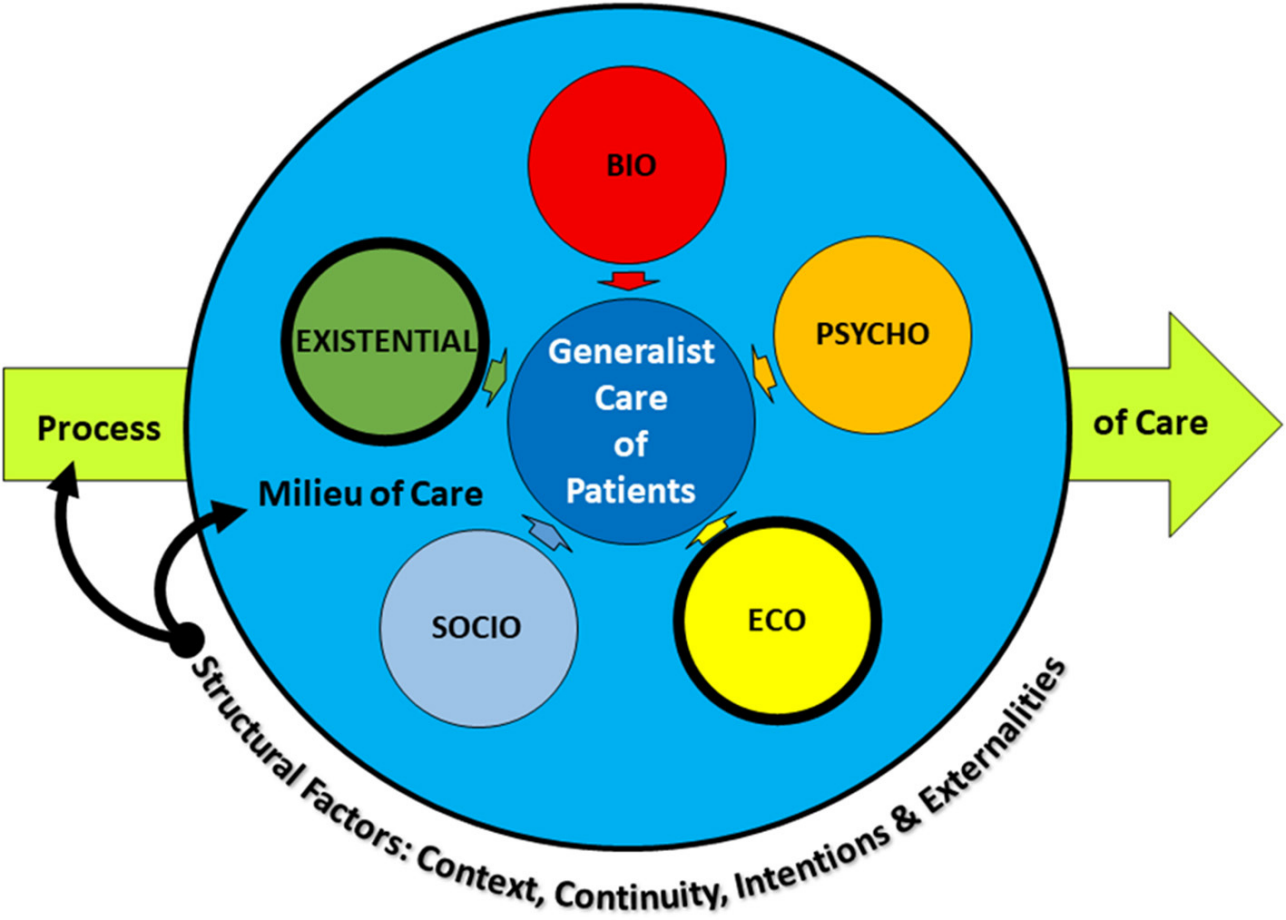
Add-ons to the traditional BPS approach in generalist practice.

In addition, add-ons can take the form of specific structural factors that affect the milieu in and the process by which practitioners interpret the BPS approach (Figure 1). Differences in these factors invariably alter how individual practitioners implement the approach. These specific factors reflect the location and setting of care, the nature of any particular patient's concerns, and the characteristics of the practitioner's professional background (53). Drawing from literature that speaks to the nature of generalist practice—specifically, that generalists are likely to see patients across the lifespan in short visits over long periods of time; attend to concerns both acute and chronic; strategize care that simultaneously bridges prevention, management, and cure; and address multiple undifferentiated problems across a range of concerns (54)—we suggest four structural factors are key: context, continuity, intentions, and externalities (Table 2).

**Table 2:** BioPsychoSocial add-ons: structural factors.

**Factors**	**Rationale**
Context	Context is the medium in and through which all relationships exist, be they professional (as between a practitioner and patient) or personal (as in activities of daily life). Appreciating the social, cultural, and emotional influences of context on clinical encounters is helpful when assessing how patients (and, reciprocally, practitioners themselves) make sense of disease and illness. Contextual factors can be as simple as asking a patient trying to quit smoking about other tobacco users in the household. Alternatively, they can be as complex as trying to appreciate how Adverse Childhood Experiences (ACEs) or Adverse Shared Historical Experiences (ASHEs) influence patients' presentations and practitioners' responses (55, 56). As in the case of the current COVID-19 pandemic, contextual factors can also arise up without much warning and trigger physical, emotional, and relational distress around the globe.
Continuity	Interactions between practitioners and patients often evolve over a lifetime. It is common for patients in generalist practices to see the same practitioner over the course of several encounters for concerns of varying clinical intensity. Continuity of relationship allows for the evaluation, diagnosis, and management of emerging concerns in light of the natural history of diseases and individual differences in their expression (57). Evidence shows that continuity of care and relationships lead to better patient experience and improved health outcomes (58). Continuity also positively influences practitioners' attitudes toward their work. Multiple studies document that the most meaningful aspect of doctoring is developing and maintaining interpersonal relationships (59).
Intentions	The elements of therapeutic communication—including, but not limited to, active listening (60), the demonstration of empathy (61), and a probe-and-pause question-oriented approach (62)—are habits of practice that contribute to improved patient outcomes (63). So are intentions of practice: the thoughtful consideration of how to develop and employ one's self-awareness and relational acumen as a practitioner (64). Specifically, the BPS approach neither starts nor stops at the exact moment an exam room door opens or closes: its use is conditioned by practitioners' attention to the iterative steps of recognition, engagement, reflection, action, and review in their therapeutic interactions (65), the ongoing, in-the-moment process of being with patients in clinical encounters.
Externalities	Externalities are commonly encountered factors outside the practitioners and patients' control that shape their interactions. They include reimbursement schemes that preferentially reward throughput over humanistic care (66); in-room electronic medical record systems that divert attention from direct care of the patient (67); and educational systems that prioritize narrowly biomedical models of diagnosis and treatment to the exclusion of social and emotional determinants of care (68). Absent acknowledgment, consideration, and action, in the face of such externalities care may become overly transactional and symptom focused, leading to poorer overall outcomes for patients and reduced job satisfaction for practitioners.

## Adding-in to the BPS Approach

The BPS approach initially focused on individual patients embedded in complex bureaucratic systems. We agree this perspective is important. We also suggest that generalist practitioners develop the ability to see themselves as integral parts of these systems. We encourage them to appreciate their use of the BPS approach with patients as a means of identifying add-ins—organically produced insights that arise in the course of patient encounters—in order to critically consider how to do the best they can, in any moment at hand, given the circumstances of any clinical situation, and help patients move toward health.

Given the current culture of medicine that marginalizes the holistic practice of generalist medicine, attending to these tasks may not be easy. We suggest, however, the BPS approach is bi-directional, and that by applying it with patients in everyday practice generalists can develop their professional identities as caring and humanistic healers. They can come to understand how clinical encounters are coproduced (69), examine how practitioners' own implicit biases influence the provision of care as well as the healing process (70), and consider how personal histories and professional socializations affect the processes and outcomes of care (71). Additionally, they can appreciate how to employ cultural sensitivity (72), with cultural humility (73), relative to patients and their concerns and as influenced by where they practice and the resources available. They can learn how to recognize, investigate, and manage the feelings and thoughts that are integral to enhancing practitioner equanimity in the face of anxiety and contentment in the face of stress.

Adding-in the BPS approach, with the aim of strategically cultivating professional growth, calls for generalist practitioners to use other key principles of practice [including such longstanding principles as affinity, intimacy, curiosity, and fidelity (74)] in the moral and ethical milieu that exists between them and their patients (75). It encourages them to nurture attributes such as emotional intelligence (76), adaptive expertise (77), and clinical courage as instruments of therapeutic change (78). It also encourages generalists to engage in the communities they serve (79), to use and cultivate an anthropological gaze as to the world around them (80), and to see their role as a call to action for social accountability (81). The BPS approach, in this way, is an expression of the interconnected nature not only of the doctor-patient relationship, but also a guide for generalists to become more adept—clinically wise—on their professional path from competency to capability to capacity and beyond (82).

**Figure 2:**
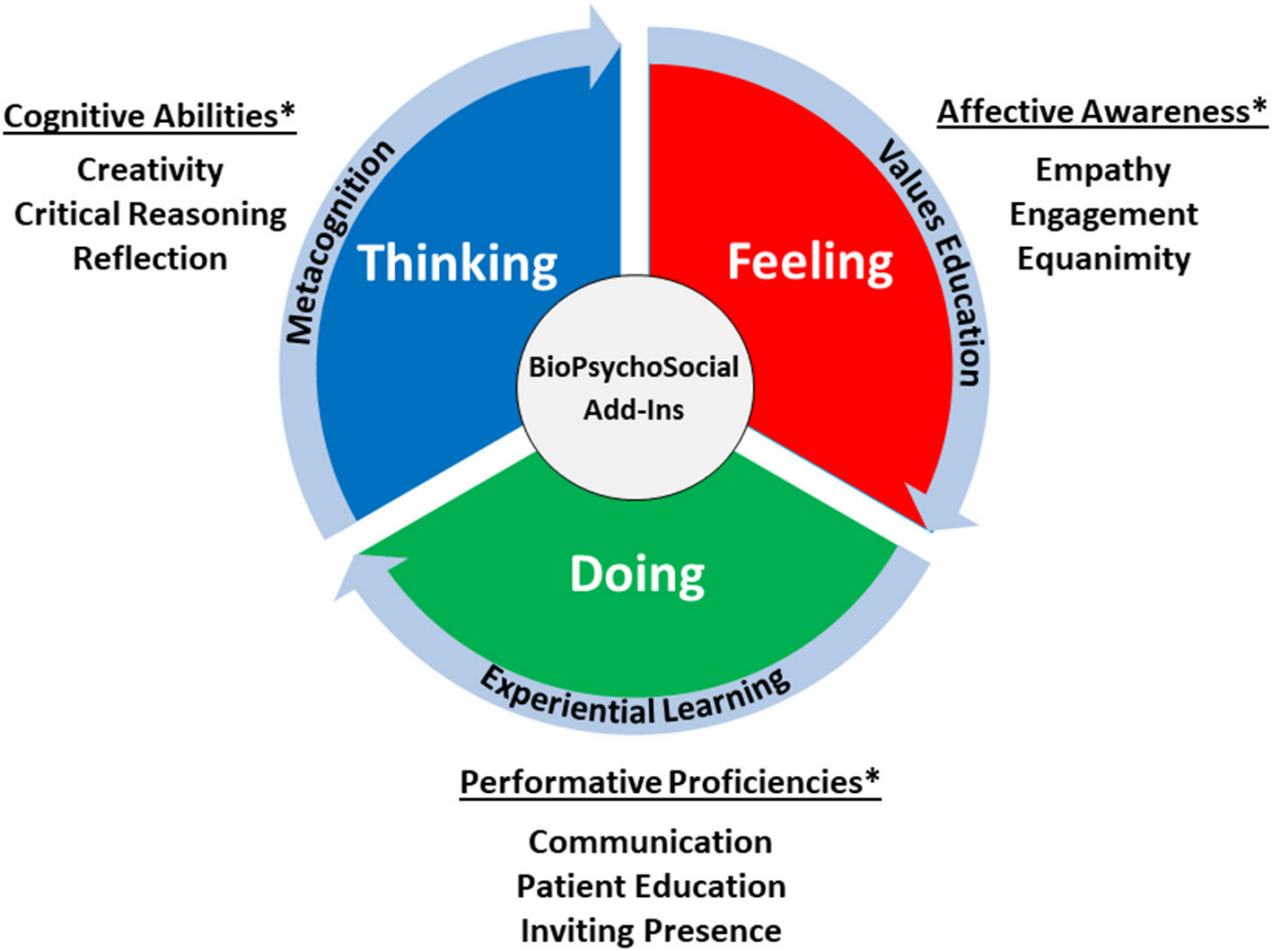
Developing clinical wisdom—dimensions of personal growth. *We list examples in these categories for illustrative purposes only; they are not all-inclusive in nature.

Generalists (and, indeed, specialists, subspecialists, and other health care professionals) can consciously develop their clinical wisdom by attending to add-ins as personalized insights into growing their professional identities (83). This growth emerges from thinking about thinking (metacognition) (84), feeling (values education) (85), and doing (experiential learning) (86). In turn, such reflective thinking can help generalists enhance their cognitive abilities, expand their affective awareness, and develop their performative proficiencies (Figure 2).

## Clinical Case Study

We present a brief clinical case study to illustrate how add-ons and add-ins are intermingled with the process of applying a BPS approach in clinical encounters (Table 3). For demonstration purposes only, we list both add-on themes and factors and add-in insights separately and sequentially; the reality is that the BPS approach and the themes, factors, and insights we enumerate are more dynamic than static and more systemic than linear in nature. As many generalists have previously noted, using this kind of approach is a “way of being” in practice that is incorporates practitioner awareness, patient- and relationship-centeredness, in-the-moment flexibility, integrated teamwork, and shared presence as regularly practiced habits (11).

**Table 3:** Clinical case study—patient presentation, add-ons, and add-ins^*^.

**Patient presentation^†^**	**Add-Ons^‡^**		**Add-Ins^§^**
The patient is a 63-year-old male who presents with several years of headaches, dizziness, and unsteadiness. He recently arrived in the U.S. as a religious refugee from Moldova. His wife accompanies him; a Russian-speaking interpreter translates.	Biological Ecological Context	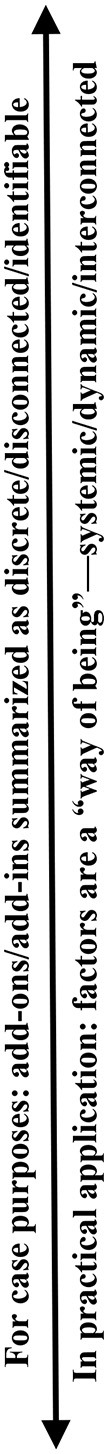	Thinking: Reflection
He notes he is ashamed by his unsteadiness—in his rural community of origin, he was considered the town drunk. “Only I don't drink”, he notes. “I am a Christian. My children are now here, in the U.S. I want them to know I am a good father.”	Psychological Existential Social		Feeling: Empathy
The patient's blood pressure is 240/140, his pulse 100. I then “talk” him through his exam. His heart sounds are regular with a normal S1 and S2. His lung fields are clear. He has trace lower extremity edema. He is alert and oriented. His neurological exam is non-focal.	Biological		Doing: Communication
I ask if he has ever heard of high blood pressure; he has not. I explain how his blood pressure might be the sole cause of his symptoms. I explain I will order some lab tests, get a tracing of his heart (EKG), and suggest some pills for him to take daily. I note my medical assistant and I will see him, in short visits, frequently, over the course a month and regularly thereafter. I inquire, “How does this sound to you?” I ask his wife, “Are you, too, comfortable with this plan? Do you have other concerns that we haven't addressed?”	Biological Biological Social Externality Ecological Intention		Thinking: Critical Reasoning Doing: Education Feeling: Engagement Doing: Inviting Presence
I request the interpreter investigate what the patient and his wife have understood and leave the room to see another patient. I return after labs are drawn and an EKG done to prescribe a standard antihypertensive medication.	Externality Biological		
At a visit six month later, the patient's blood pressure controlled with multiple medications and his dizziness and unsteadiness fully resolved, the patient—very appreciative for the care we have provided—asks, “now that I am cured, can I stop my pills?”	Continuity Biological Psychological Existential		Feeling: Equanimity Thinking: Creativity

* *This clinical case presentation summarizes actual interactions that occurred in Dr. Ventres' community-based practice*.

† *For a more detailed review of this case study, please see Ventres (62)*.

‡ *We note only add-ons mentioned in the text (Tables 1, 2). We encourage practitioners to use these as a starting point for further personalizing the BPS approach*.

§ *We use a thinking, feeling, and doing model to frame add-ins to the BPS approach. Other learning processes could function as alternative methods of self-growth, including the questioning list noted in Ventres (62)*.

## Discussion

The point of introducing these considerations is to suggest that generalist practitioners consider the BPS approach not as a model set in stone, but as (1) an inspiration for further integrating BPS concepts into practice, (2) a stimulus to promote patient- and relationship- centered approaches to patient care, and (3) a means to of repositioning themselves in the space between patients' lived experience and the culture of medicine (87). The BPS approach offers generalists not only a broad understanding of the many factors that contribute to the evaluation, diagnosis, and management of presenting problems, but also a path to reconceptualize professional growth in service of therapeutic agency (one's ability to affect positive change) and well-being on both sides of the stethoscope.

More important, the point is that generalist practitioners consider the BPS approach as a template for exploring their own contributions to the healing process by examining not only their roles and relationships vis-à-vis the patients they serve, but also the attributes of clinical wisdom they develop and express along the course of their professional lives. The add-ons and add-ins we suggest can and should be modified or supplemented by others considerations, as appropriate—the overriding goal is doing the right thing at the right time, under the circumstances at hand, for the betterment of patients' health and with the intent of improving their well-being. The BPS approach in this way can help generalists envision, create, and incorporate original add-ons and add-ins to enrich their healing talents.

In fact, we encourage generalist practitioners to take personal ownership of the BPS approach and apply it, distinctively, with all patients in their daily work. We hope they use the approach as a directional marker pointing the way toward individual clinical excellence in holistic patient care. Collectively, we hope they and their colleagues in other disciplines use it as guide to making the practice of generalist medicine and medicine as a whole more inclusive, humane, efficacious, and satisfying. Given current circumstances, external incentives tying compensation to patient experience may be helpful in nudging these aspirations along (88).

These aspirations are particularly fitting as means of countering the increasingly fragmented, hyper-technical, production-oriented, industrialized model of medical practice that exists at this very moment in time, especially in the United States. The BPS approach may also help remediate traditional medicine's acknowledged failures in the face of injustice, inequity, and political polarization, forces that increasingly appear to negate not only the ultimate effectiveness of medicine, but also the healing satisfaction characteristic of its practice.

## Further Thoughts

First, we are fully aware, and have noted above, that the BPS approach is applicable beyond generalist medicine. The approach has utility in specialty and subspecialty medical practice as well as in a variety of other health care disciplines, and literature emergent from those disciplines has enlightened our own understandings of the BPS approach. Our purpose in focusing on generalist practice is not to exclude others who attend to patients. It emerges, however, from our assessment that the BPS approach is central to the everyday practice of generalist medicine: with the exception of those patients who present with imminently life threatening conditions, the BPS approach is applicable, to greater or lesser degree, at all times in all situations with all patients who present to generalist practitioners. Due to the nature of clinical interactions in specialty and subspecialty care, the BPS approach is generally—and appropriately—a supplement to the biomedical model, invoked either in response to certain presenting problems or when the traditional linear course of diagnoses and treatment has been tried and failed.

Second, any approach to understanding the complexity of human life in relationship to the very real experience of disease, illness, and sickness will inherently find itself limited by the words used to describe it. This is especially true when considered independently of the context of a particular patient's individual history, current experience, or the circumstances under which individuals turn to the medical care system in times of need. No textual explanation or graphic representation can wholly represent the dynamic process of clinical encounters (36), just as no single recommendation for enhancing such encounters is applicable or appropriate in each and every setting. The reality is that the BPS approach, with or without add-ons or add-ins, can only approximate some of what goes on between practitioners and patients (89), let alone what goes on in the consciousness of individual patients or practitioners beyond the veil of clinical presentations.

Third, another reality is that interactions between practitioners and patients do not always go as planned or go well. No conceptual approach or practiced skill can guarantee perfection in all clinical encounters, especially in light of the many influences that shape them. While challenging to endure, conflicts and mistakes can provide generalist practitioners with opportunities to learn and incorporate new knowledge, new patterns of thought, and new expressions of care in their work with patients. Often it is not what one does, but what one does next that counts—communication strategies such as conversational repair and apology can be taught, learned, and put into practice, benefitting patients, practitioners, and the therapeutic relationships that exist between them (90, 91).

## Conclusion

The BPS approach has been a part of the practice and teaching of generalist medicine since its introduction over fifty years ago. It provides an important foundation for considering, conducting, observing, reflecting upon, and providing feedback about the intricacies of clinical care, healing interactions, and practitioner-patient communication. It has, however, struggled to gain broad acceptance in the face of a dominant linear model of biomedical practice. Given new developments in the practice of generalist medicine and the world as we know it, we suggest that generalist practitioners view the BPS approach and its offspring, Patient- and Relationship-Centered Care, as dynamic and modifiable templates in service of both addressing patient concerns and improving their own clinical awareness. We offer for reflection ways to add-on to the BPS approach several thematic considerations and structural factors in order to further develop its efficacy with patients. We also suggest how generalists can use the BPS approach as an add-in to enhance self-awareness and understand their own signature presence as healing professionals. We encourage generalist practitioners to view the BPS approach as an invitation to explore ways to improve patients' health and well-being as well as their own joy and resilience in the practice of medicine.

## Author Contributions

WBV and RMF contributed to the conception and design of this manuscript. WBV wrote the first draft of the manuscript. WBV and RMF contributed to manuscript revision, read the final version of the manuscript, and approved the submission version. All authors contributed to the article and approved the submitted version.

## Conflict of Interest

The authors declare that the research was conducted in the absence of any commercial or financial relationships that could be construed as a potential conflict of interest.

## Publisher's Note

All claims expressed in this article are solely those of the authors and do not necessarily represent those of their affiliated organizations, or those of the publisher, the editors and the reviewers. Any product that may be evaluated in this article, or claim that may be made by its manufacturer, is not guaranteed or endorsed by the publisher.
